# The role of individual protein kinase C isoforms in mouse mast cell function and their targeting by the immunomodulatory parasitic worm product, ES-62

**DOI:** 10.1016/j.imlet.2015.09.001

**Published:** 2015-11

**Authors:** Kara S. Bell, Lamyaa Al-Riyami, Felicity E. Lumb, Graham J. Britton, Alastair W. Poole, Christopher M. Williams, Ursula Braun, Michael Leitges, Margaret M. Harnett, William Harnett

**Affiliations:** a Strathclyde Institute of Pharmacy and Biomedical Sciences, University of Strathclyde, Glasgow G4 0RE, UK; b School of Medical Sciences, University of Bristol, Bristol BS8 1TD, UK; c The Biotechnology Centre of Oslo, University of Oslo, Oslo 0349, Norway; d Centre for Immunobiology, Glasgow Biomedical Research Centre, Institute of Infection, Immunity and Inflammation, University of Glasgow, Glasgow G12 8TA, UK

**Keywords:** ES-62, Mast cell, PKC, Immunomodulation, Signal transduction

## Abstract

ES-62, a glycoprotein secreted by the filarial nematode *Acanthocheilonema viteae*, has been shown to modulate the immune system through subversion of signal transduction pathways operating in various immune system cells. With respect to human bone marrow-derived mast cells (BMMCs), ES-62 was previously shown to inhibit FcϵRI-mediated mast cell functional responses such as degranulation and pro-inflammatory cytokine release through a mechanism involving the degradation of PKC-α. At the same time, it was noted that the worm product was able to degrade certain other PKC isoforms but the significance of this was uncertain. In this study, we have employed PKC isoform KO mice to investigate the role of PKC-α, -β -ϵ, and -θ in mouse BMMCs in order to establish their involvement in mast cell-mediated responses and also, if their absence impacts on ES-62’s activity. The data obtained support that in response to antigen cross-linking of IgE bound to FcϵRI, pro-inflammatory cytokine release is controlled in part by a partnership between one conventional and one novel isoform with PKC-α and -θ acting as positive regulators of IL-6 and TNF-α production, while PKC-β and ϵ act as negative regulators of such cytokines. Furthermore, ES-62 appears to target certain other PKC isoforms in addition to PKC-α to inhibit cytokine release and this may enable it to more efficiently inhibit mast cell responses.

## Highlights

• Knock out mice have been used to explore the role of PKCs in mast cell (MC) function.
• Individual PKC isoforms may positively or negatively regulate MC cytokine responses.
• Certain PKC isoforms in MCs are targeted by the parasitic worm product ES-62.

## 1 Introduction

The Protein kinase C (PKC) family comprises a number of phospholipid-dependent serine/threonine protein kinases that play critical roles in signal transduction pathways within a cell, controlling fundamental functions such as cell growth, proliferation, differentiation and apoptosis [1], [2]. Currently, up to 10 PKC isoforms have been identified in mammals, and have been shown to have extensive tissue distribution and cellular localization [1], [3]. PKC family members are divided into three main subsets based on their structure and lipid requirements for activation: (a) calcium and diacylglycerol (DAG)-regulated conventional isoforms (cPKCs: α, βI, βII and γ); (b) calcium-independent, DAG-dependent novel isoforms (nPKCs: δ, ϵ, η, and θ); (c) the atypical isoforms which are both calcium and DAG independent (aPKC: ζ and ι/λ) [3], [4], [5], [6], [7], [8], [9].

Mast cells constitute a heterogeneous cell population that exhibits distinct phenotypic and functional properties [10], [11], [12], with differences in PKC isoform expression being reported to exist both between species and in mast cell subtypes within a species [13], [14], [15]. For example, the rat mast cell line RBL-2H3 expresses a set of PKC isoforms distinct from that of mouse bone marrow-derived mast cells (BMMCs) and human mast cells (HMCs) [13], [14], [15]. Conventional PKC isoforms have been implicated in differential mast cell responses such as degranulation and the release of pro-inflammatory mediators like cytokines, prostaglandins and leukotrienes [14], [15], [16]. Thus, in the context of antigen stimulation, genetic knockout studies have demonstrated the importance of PKC-β in mast cell degranulation [16] whilst PKC-α has been implicated as playing a critical role in the production of cytokines [14]. It is the release of such pro-inflammatory mediators following the activation of mast cells via cross-linking of FcϵRI that results in the pathological consequences of mast cell activation and is largely responsible for the symptoms associated with allergic disorders [11], [17].

The phosphorylcholine (PC)-containing secreted glycoprotein, ES-62, derived from the filarial nematode *Acanthocheilonema viteae* has previously been shown to possess immunomodulatory properties via subversion of signal transduction pathways operating in various immune system cells such as macrophages, B cells and dendritic cells [18] and reflecting this, it exhibits therapeutic potential in models of inflammatory disease such as asthma, contact hypersensitivity and rheumatoid arthritis [19]. In addition, ES-62 has been shown to inhibit human mast cell function and consequent allergic responses by selectively targeting key signals involved in degranulation and cytokine release such as calcium mobilization and the expression of PKC-α [13]. Likewise, ES-62-mediated down-regulation of PKC-α has been demonstrated in a range of mouse mast cell subtypes (bone marrow-derived mucosal-type mast cells; BM-connective tissue mast cells and peritoneal-derived mast cells) and downregulation of PKC-δ in BMMCs, ultimately rendering the cells hypo-responsive to subsequent stimuli [10]. An additional finding in the human mast cell study was that ES-62 reduced the expression level of PKC isoforms other than PKC-α, namely PKCs -β, -δ, -ι and –ζ [13] although the significance of this for ES-62's immunomodulatory activity is uncertain. The aim of this study was thus to investigate the role of various PKC isoforms (α, β, ϵ, θ) in BMMC functional responses through the use of PKC-isoform deficient (PKC isoform^−/−^) mice and to determine whether the absence of a particular isoform impacts on ES-62 activity.

## 2 Materials and methods

### 2.1 Mice and reagents

Both male and female BALB/C mice aged between 6 and 8 weeks old that had been bred and maintained at the University of Strathclyde animal unit were used to generate bone-marrow derived mast cells (BMMCs) for certain aspects of this study. Additionally for PKC isoform^−/−^ studies, PKC-α (C57BL/6-Sv129 background), -β (129/Sv/129/Ola background), -θ (B10.PL background) and –ϵ (C57BL/6Jax background) deficient mice in conjunction with their age and sex matched wild-type controls were used to generate BMMCs. PKC-α^−/−^ and PKC-θ^−/−^ mice were generated as previously described [20], [21]. Each set of PKC knockout (KO) mice and their wild-type (WT) counterpart were bred and maintained at the specific laboratory group's animal unit before the bones were harvested and shipped to the University of Strathclyde. It should be noted that we observed variability in the stimulated cytokine response of mast cells derived from the different WT control mice: their different origins and facilities they were raised in, in addition to differences in genetic background, presumably represent likely contributors to this. All animals used were pathogen-free and all procedures were conducted in accordance with Home Office, U.K. animal guidelines and with the approval of the local ethical committees.

### 2.2 Bone marrow-derived mast cells (BMMCs)

Intact femur and tibia bones were dissected from mice or shipped from collaborators on ice within 24 h and soaked in 70% ethanol to remove fibroblasts and connective tissue before the ends of each femur and tibia were snipped and single cell suspensions made from the bone marrow by flushing cold RPMI complete (RPMI 1640 with 10% HI FCS, 2 mM l-glutamine, 100 U/mL Penicillin and 100 μg/mL streptomycin) through one end of the bone with a 23 gauge sterile needle. The resulting cell suspension was then mixed gently using a 22 gauge sterile needle attached to a syringe to remove any clumps of cells before passing the cell suspension through a sterile BD sieve. The mixture was then centrifuged at 400 g for 5 min before the supernatant was discarded and the remaining cell pellet was re-suspended in fresh culture medium and cells counted using a haemocytometer. BMMCs were derived by culture of bone marrow progenitors at 0.5 × 10^6^/mL in RPMI with 10% HI FCS, 2 mM l-glutamine, 100 U/mL Penicillin, 100 μg/ml Streptomycin, 1 mM sodium pyruvate, 10 mM HEPES, and 50 μM β-mercaptoethanol supplemented with conditioned growth medium from KLS-C (1%; SCF) and TOP3 (3%; IL-3) cell lines. Cells were incubated at 37 °C/ 5% CO_2_ for 3–6 weeks in total. BMMCs were counted and provided with fresh medium and cytokines and transferred to a fresh flask twice a week. After 3–4 weeks maturation, the cells were tested for their expression of the cell surface markers c-kit, FcϵRI to ascertain their identity as mature mast cells.

### 2.3 Mast cell phenotyping by flow cytometry

BMMCs were phenotyped by flow cytometric analysis and the presence of the cell surface markers, c-kit and FcϵRI, were confirmed. Briefly, cells were counted (0.1 million cells per sample), washed 1X with cold PBS and re-suspended in 50 μL of FACS buffer (PBS containing 2% BSA (W/V) and 2 mM EDTA) per sample. The aliquots of cells were then incubated with 50 μl of fluorescently-tagged antibodies to confirm the presence of the cell surface markers for 30 min in the dark on ice. After labelling, cells were washed a final time with 3 ml FACS buffer at 400 g for 5 min before being re-suspended in 250 μl of FACS buffer for flow analysis using a Becton Dickinson BD FACS CANTO™ flow cytometer. Analysis was performed on a minimum of 10, 000 events. Flowjo software (Tree Star Inc., OR, USA, version 7.6.3) was used for final analysis of the flow cytometry data.

### 2.4 Mast cell stimulation

Unless otherwise stated, mast cells were plated at 1 × 10^6^cells/mL on a 12 well sterile tissue culture plate. Cells were then sensitized with anti-DNP IgE (0.5 μg/mL) in the presence or absence of ES-62 (2 μg/mL) or ES-62 alone. Control wells were incubated with medium alone (RPMI complete BMMC medium supplemented with growth factor media 3% TOP3 and 1% KLSC). Cells were incubated for 24 h at 37 °C/5% CO_2._ Following this, samples were collected into 1.5 mL sterile eppendorf tubes and centrifuged at 500 g for 5 min. The supernatant was then removed and the cells washed twice with sterile PBS (by centrifuging at 500 g for 5 min). Cells were then re-suspended in 1 mL of BMMC complete medium supplemented with 3% TOP3- and 1% KLSC-conditioned medium.

500 μL of cells were then transferred to the appropriate well in a 24 well plate forming a Basal (unstimulated) and a stimulated well for each experimental condition. Cells were then stimulated by addition of medium alone, 0.5 μg/ml of DNP-HSA to cross-link FcϵRI or 1 μg/ml of LPS (*Salmonella Minnesota;* Sigma–Aldrich, Dorset, U.K.). Cells were incubated for 24 h at 37^°^C, 5% CO_2._ Reactions were terminated after the desired culture period by centrifugation at 500 × *g* for 5 min and supernatants aspirated for determination of cytokine release. The resultant supernatant was stored at –20 °C until required.

### 2.5 Cytokine analysis

Cytokine ELISAs for IL-6 and TNF-α (limits of detection 15 pg/mL for both; BD Biosciences) were performed on triplicate samples according to the suppliers’ recommendations and developed using KPL Sureblue, TMB Microwell Peroxidase Substrate. The reaction was terminated by stop solution, 2N H_2_SO_4._ The final reactions were measured at 405 nm in an ELISA microplate reader.

### 2.6 Western blotting

Mast cells were washed in cold PBS by centrifugation (400 ×* g*; 5 min) to terminate any reactions and/or to remove serum before cell lysis. Untreated or stimulated cells (as described previously) were lysed by the addition of 20–40 μL (cell number dependent) of modified Radio-Immunoprecipitation Assay (RIPA) buffer (RIPA Buffer from SIGMA, USA). Modified RIPA buffer consisted of 100X HALT™ Protease and Phosphatase Inhibitor cocktails (Thermo Scientific, IL, USA) diluted 1 in 100 in RIPA buffer. After re-suspending the cell pellet in modified RIPA buffer, cells were incubated on ice for 15 min to allow solubilization before centrifugation at 5000 × *g* for 10 min. The resultant supernatants containing the cell lysates were stored at −80 °C for long-term storage or −20 °C for short-term storage before being analysed by SDS-PAGE gel electrophoresis and Western Blot analysis.

Equal protein loadings of cell lysates or control extracts (30–40 μg per lane for BMMCs unless stated otherwise) determined by BCA protein assay (bicinchoninic acid, Thermo Scientific Pierce) were resolved using XCell *surelock* Mini-cell kit with NuPAGE^®^ Novex^®^ high-performance precast 4–12% Tris–Bis gels and NuPAGE^®^ buffers and reagents (Invitrogen). Proteins were then transferred onto a Nitrocellulose membrane (Amersham) using NuPAGE^®^ transfer buffer supplemented with 10% methanol and successful protein transfer was validated by Ponceau Red staining. Membranes were then washed in Tris–buffered saline (TBS) (0.5 M NaCl and 20 mM Tris, pH 7.5) with 0.1% (v/v) Tween-20 (TBS/Tween) and non-specific binding sites blocked for one hour in TBS-Tween containing 5% non-fat milk protein (Marvel). Membranes were then incubated with the appropriate primary detection antibody overnight at 4 °C on a shaker. All antibodies tested were either diluted in 5% non-fat milk or 5% BSA. Following primary antibody incubation, nitrocellulose membranes were washed as before and incubated in the appropriate HRP-conjugated secondary antibody (diluted up to 1: 2500 in wash buffer containing 5% non-fat milk) for 1–2 h at room temperature. Nitrocellulose membranes were then washed as before and protein bands visualised using the NOVEX^®^ ECL HRP Chemiluminescent detection system (Invitrogen) and UltraCruz™ Autoradiography film (Santa Cruz biotechnology Inc, USA). Densitometry was performed using Image J software.

### 2.7 Statistical analysis

The data presented are representative of the stated number of individual experiments involving the indicated statistical analysis using Graphpad Prism software. The statistical analysis for cytokine production was performed in Graphpad PRISM using either a one way ANOVA or a two way ANOVA with Bonferoni post test where *P < 0.05 **P < 0.005 *** *P *< 0.001 **** *P *< 0.0001.

## 3 Results

### 3.1 The effect of PKC-isoform deficiency on maturation of bone marrow-derived mast cells (BMMCs)

**Fig. 1:**
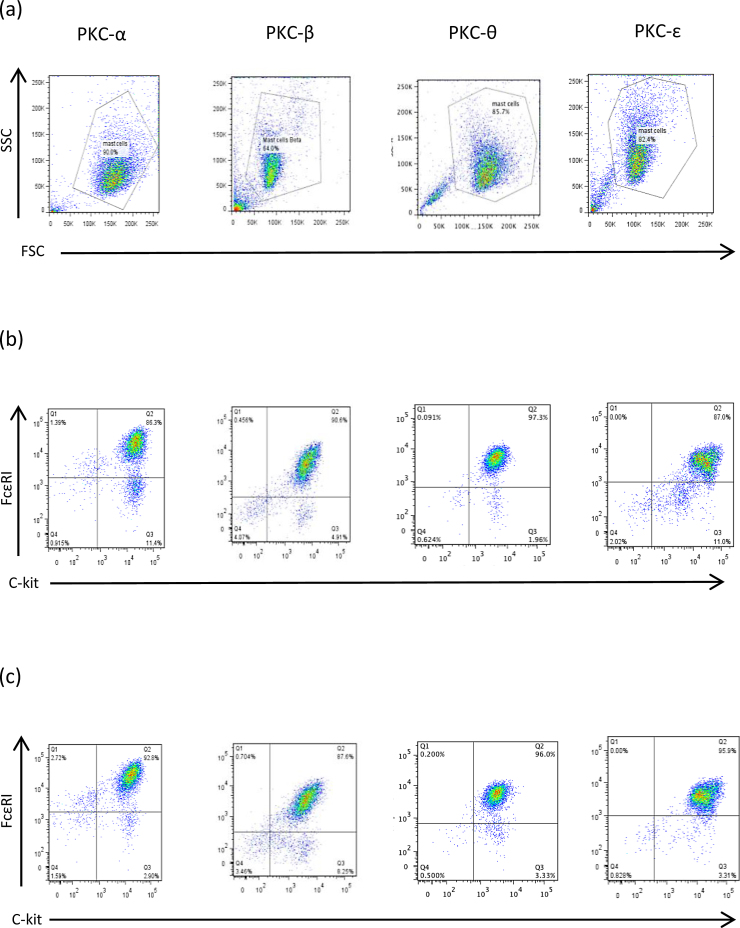
Phenotyping of BMMCs from PKC isoform^−/−^ and matched WT culture are shown in (a)–(c). Forward scatter (FSC) versus side scatter (SSC) parameters of KO BMMCs allowing gating of live cells on the basis of their size and granularity, respectively (a). Mast cells gated as described in panel (a) were examined for their expression of c-kit and FcϵRI (relevant to appropriate isotype controls; data not shown) for both PKC isoform WT (b) and PKC isoform ^−/−^ (c) cultures.

Following the in vitro development of BMMCs from various PKC isoform^−/−^ and matched WT mice*,* cells were examined for co-expression of the mast cell surface markers, c-kit and FcϵRI as an indication of their status as mast cells. Such analysis (Fig. 1 and Table 1) suggested that the absence of any of these PKC isoforms individually does not prevent maturation of this subtype of mouse mast cells from bone marrow progenitors in vitro.

### 3.2 The effect of PKC-isoform deficiency on FcϵRI-mediated cytokine production by BMMCs

**Fig. 2:**
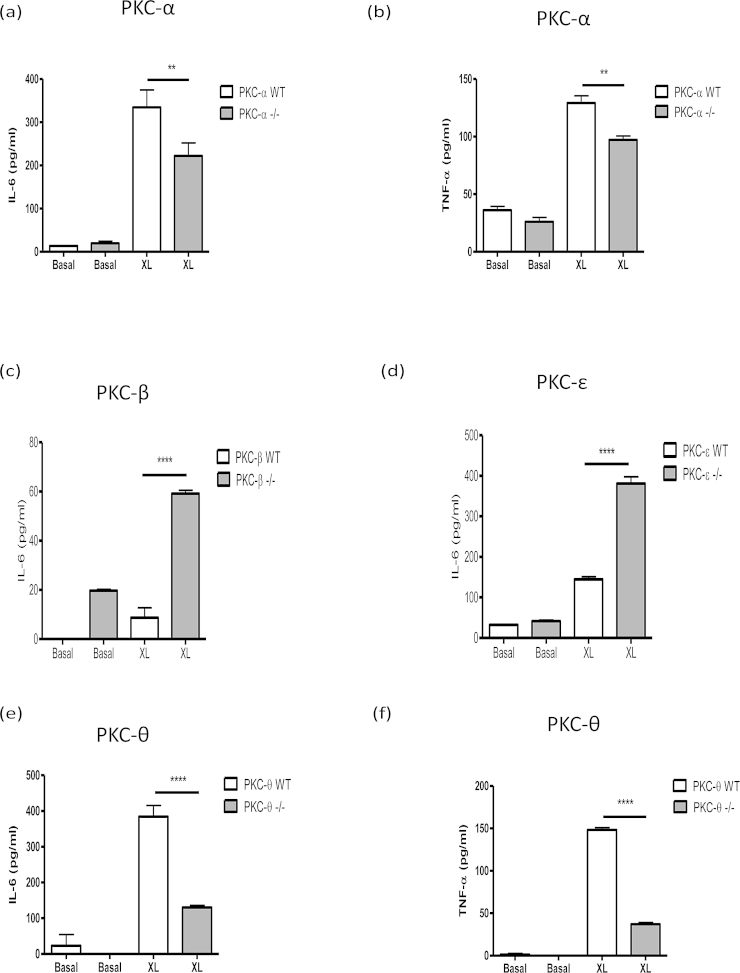
FcϵRI-mediated cytokine release by PKC isoform^−/−^ and matched WT BMMCs. BMMCs were sensitized with murine anti-DNP IgE antibodies (IgE; 0.5 μg/mL) for 24 h then stimulated with DNP-BSA to induce cross-linking (XL) for 24 h. Cell culture supernatants were harvested and cytokine levels of IL-6 and TNF-α release were measured by ELISA from PKC isoform-deficient and corresponding WT cells: PKC-α (a and b), PKC-β (c), PKC-ϵ (d) and PKC-θ (e and f) (Means ± SD, *n* = 3). The data presented are single experiments representative of at least 3 independent experiments apart from those involving PKC-α^−/−^ and PKC–β^−/−^ IL-6 release, which are representative of 2 individual statistically significant experiments. Statistical analysis was by ANOVA with Bonferoni post test where *P < 0.05 **P < 0.005 ***P < 0.001 ****P < 0.0001.

By contrast, these PKC isoforms appear to play important roles in the regulation of pro-inflammatory cytokine secretion by BMMCs in response to cross-linking of bound IgE antibodies by antigen: for example, PKC-α^−/−^ BMMCs produced significantly less IL-6 and TNF-α, in comparison to their WT counterparts (Fig. 2a and b; summarised data values in Table 2). Interestingly therefore, although there appeared to be very little IL-6 release following FcϵRI cross-linking in BMMCs from the matched littermate WT mice, deficiency in the PKC-β isoform resulted in an increase in antigen-stimulated IL-6 secretion (Fig. 2c; Table 2). Similarly, analysis of PKC-ϵ^−/−^ and WT BMMC following antigen cross-linking of IgE revealed that IL-6 production is increased by deficiency in PKC-ϵ (Fig. 2d; Table 2). Unfortunately, no TNF-α production could be detected in any of the experiments using matched WT and PKC-β^−/−^ (*n *= 4) or PKC-ϵ^−/−^ (*n* = 3) mice following antigen cross-linking (data not shown). However, as with PKC-α deficiency, it was found that IL-6 and TNF−α secretion were significantly decreased in PKC-θ^−/−^ BMMCs following IgE cross-linking by antigen (Fig. 2e and f; Table 2) with indeed, the loss of this novel isoform having a greater impact than that of absence of PKC-α.

Collectively these data suggested that whilst PKC-α and θ promote FcϵRI-mediated IL-6 production, this is suppressed by PKC-β and -ϵ suggesting the potential for reciprocal counter-regulation of FcϵRI-mediate cytokine release from BMMCs by these PKC isoforms.

### 3.3 The effect of PKC-isoform deficiency on TLR4-mediated cytokine production by BMMCs

**Fig. 3:**
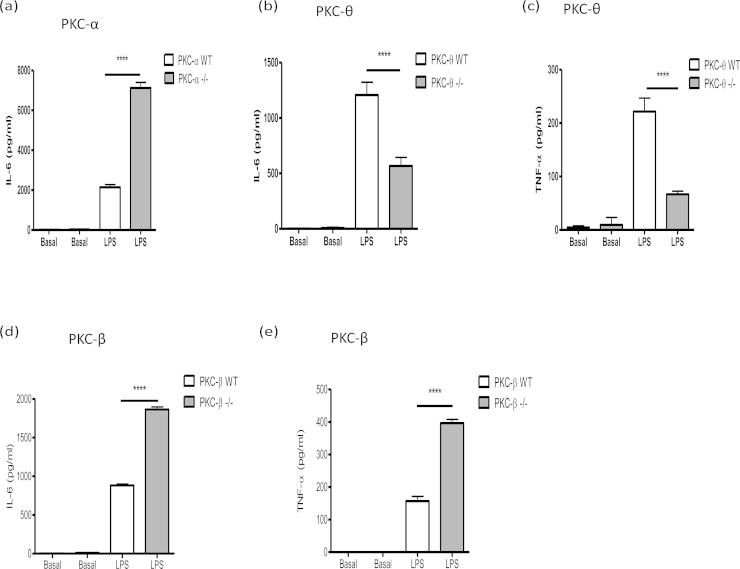
TLR-4-mediated cytokine release by PKC isoform^−/−^ and matched WT BMMCs (a: PKC-α; b and c: PKC-θ; d and e: PKC-β). BMMCs were incubated with LPS (1 μg/ml) for 24 h. IL-6 and TNF-α release were measured by ELISA. The data presented are single experiments representative of at least 3 independent experiments with the exception of those involving PKC-θ^−/−^ TNF-α release, which are representative of 2 experiments. Statistical analysis was by ANOVA with Bonferoni post test where *P < 0.05 **P < 0.005 ***P < 0.001 ****P < 0.0001.

Consistent with our previous report that various mast cell subsets, including BMMCs, respond differentially to antigen-mediated cross-linking of bound IgE (via FcϵRI) and LPS (via TLR4)-stimulation [10], PKC isoform deficiency exhibited differential effects on LPS versus antigen-stimulated cytokine production. Thus, whilst PKC-α-deficiency led to reduced antigen-stimulated IL-6 responses, the opposite trend was witnessed for LPS-stimulated production of this cytokine was significantly enhanced in PKC-α^−/−^ cells in comparison to WT cells (Fig. 3a; Table 2) suggesting that PKC-α regulation of BMMC IL-6 production was context-dependent. Moreover, PKC-α deficiency had no clear effect on LPS-stimulated TNF-α production by BMMCs (data not shown), indicating there is further differential regulation of IL-6 and TNF-α responses in such cells. Interestingly, therefore, PKC-θ-deficiency resulted in reduced LPS-stimulated IL-6 (Fig. 3b and Table 2) and TNF-α (Fig. 3c and Table 2) production indicating that this PKC isoform generally acts to promote BMMC cytokine responses and suggesting that PKC−α and −θ mediate their effects via distinct regulatory pathways. Moreover, and reflecting the proposed negative regulatory effect of PKC-β on antigen-specific responses, PKC-β-deficient BMMCs also showed significantly enhanced LPS-stimulated IL-6 secretion in comparison to WT BMMCs (Fig. 3d and Table 2) and in this case, TNF-α production was also significantly up-regulated in KO cells (Fig. 3e; Table 2) when compared to WT control: collectively, these findings suggest that PKC-β acts generally to suppress BMMC cytokine production. However, by contrast, PKC-ϵ deficiency did not substantially impact on LPS responses (results not shown), suggesting its (negative) regulatory effects are restricted to the FcϵRI pathway.

### 3.4 The ES-62 modulatory effect remains even in the absence of PKC-α

**Fig. 4:**
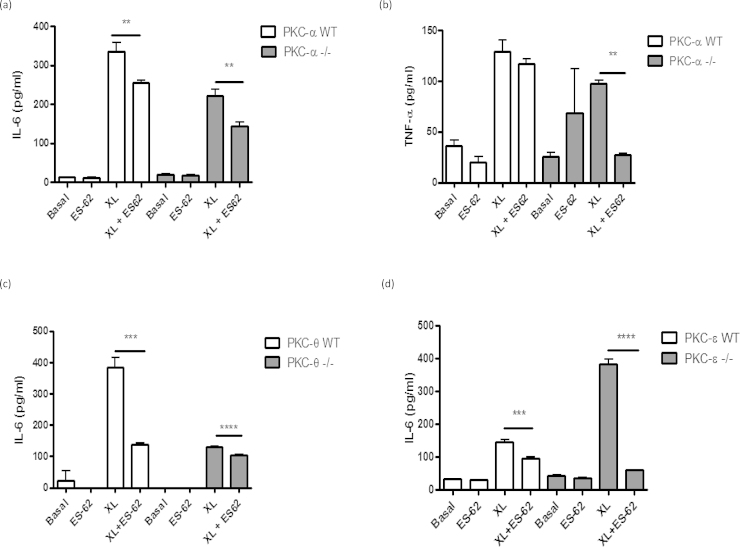
ES-62 modulatory effect in the absence of PKC-α (a and b), PKC-θ (c) and PKC-ϵ (d). PKC isoform^−/−^ and matched WT BMMCs were sensitized with 500 ng/ml anti-DNP IgE (IgE) in the presence or absence of ES-62 (2 μg/mL) for 24 h. Cells were then stimulated with 500 ng/mL DNP to induce cross-linking of FcϵRI (XL) for 24 h at 37°C. Release of IL-6 (a, c and d) and TNF-α (b) was measured by ELISA. The data presented are from a single experiment incorporating triplicate values. Statistical analysis was by ANOVA with Bonferoni post test where *P < 0.05 **P < 0.005 ***P < 0.001 ****P < 0.0001.

**Fig. 5:**
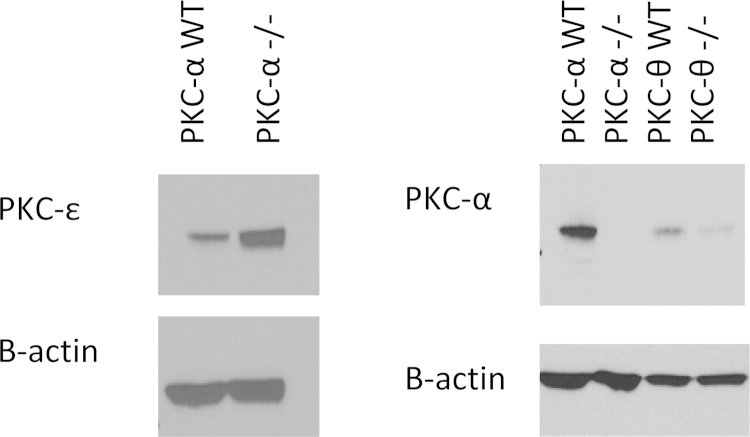
The absence of a PKC isoform can have a knock-on effect on other PKC isoform expression levels in BMMCs. Total protein extracts from PKC isoform^−/−^ BMMCs were analysed by Western Blotting for their expression of PKC-α or PKC-ϵ. The samples were also probed for β-actin as a loading control.

**Fig. 6:**
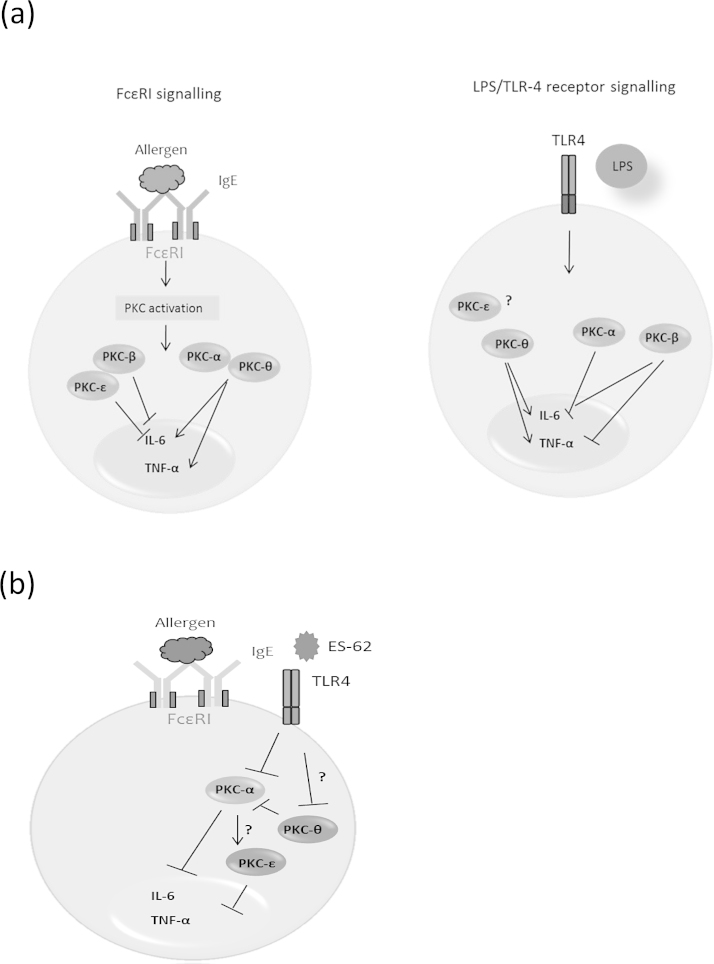
Model of differential and interactive roles of PKC isoforms in mast cells. (a) The conventional PKC isoforms α and β along with the novel isoforms ϵ and θ exhibit differential functional roles in mast cells with respect to both antigen-induced FcϵRI cross-linking and LPS stimulation. PKC-α and PKC-θ serve similar functions through promoting IL-6 cytokine secretion following antigen stimulation and this is somewhat counter-regulated by PKC-β and ϵ. The specific role of a PKC isoform also appears to be context-dependent as, for example, PKC-α acts as a negative regulator of IL-6 production in response to LPS/TLR-4 signalling yet PKC-θ positively regulates this cytokine in response to LPS. Unlike the other PKC isoforms, the role that PKC-ϵ plays in mast cell functional responses seems to be restricted to the FcϵRI pathway. (b) The signalling mechanisms whereby ES-62 inhibits mast cell function have not been fully delineated, however, we indicate that ES-62 targets key signals involved in mast cell activation such as PKC-α. Through targeting this isoform, ES-62 may also have an indirect effect on other PKC isoforms such as PKC-ϵ (a negative regulator of BMMC responses) which in turn leads to a further reduction in the secretion of pro-inflammatory cytokines. We hypothesise that ES-62 may also target PKC-θ as this isoform has not only been shown to be crucial for full mast cell activation, but it may share redundant or similar roles to PKC-α. Additionally, the expression levels of PKC-θ appear to regulate those of PKC-α and therefore through targeting this isoform, ES-62 may again be indirectly targeting a network of PKC isoforms that includes, but is not restricted to, PKC-α.

As alluded to previously, ES-62-mediated inhibition of cytokine production by human mast cells and BMMCs has been associated with PKC-α degradation following cross-linking of bound IgE receptor [10], [13], and we also noted reduced levels of PKC-α following exposure to ES-62 in the present study (results not shown). These findings are consistent with the reduced levels of antigen-stimulated IL-6 and TNF-α found in PKC-α deficient BMMCs. However, the nematode product was able to reduce IL-6 (Fig. 4a) and TNF-α (Fig. 4b) cytokine production in both WT and PKC-α deficient BMMCs following FcϵRI-mediated cross-linking, and although the reduced cytokine responses observed in the PKC-α^−/−^ cells may mimic some of the effects of ES-62, these data suggest that our previously observed down-regulation of PKC-α [13; and results not shown] is not solely responsible for the inhibitory effects of ES-62 on BMMC responses. Indeed, if anything loss of PKC-α signalling potentiates the inhibitory actions of ES-62, possibly indicating that some of the effects of ES-62 reflect indirect consequences of PKC-α deletion. Perhaps consistent with this, we have observed that ES-62 also modulates expression of other PKC-isoforms and signal transducers (e.g. PKC-δ and MyD88; 13) in BMMCs that may contribute to its induction of hyporesponsiveness in these cells. Moreover, we hypothesised that there may be some functional redundancy/adaptation of PKC isoforms such as α and θ in PKC-α^−/−^ cells that ES-62 can also target: for example, although analysis showed that ES-62 could still inhibit antigen-stimulated IL-6 production in PKC-θ^−/−^ BMMCs (Fig. 4c) this inhibition was slight, perhaps reflecting that PKC-θ deficiency alone could effectively account for the level of ES-62-mediated inhibition observed in WT cells with the residual inhibition by ES-62 in PKC-θ^−/−^ cells reflecting additional targeting of PKC-α or some other signal transducer. Moreover, whilst consistent with its proposed role as a negative regulator of FcϵRI signalling in BMMCs, PKCϵ-deficiency results in enhanced IL-6 cytokine responses that were suppressed by ES-62 (Fig. 4d): however, by contrast and perhaps surprisingly given that PKC-β appears to exhibit negative regulation of BMMC cytokine production, ES-62 inhibition of such responses was not consistently modulated by PKC-β deficiency (results not shown). We therefore investigated whether ES-62 was mediating its suppressive effects on FcϵRI-mediated cytokine responses by direct and indirect effects of PKC-α degradation by determining the effects of PKC-α deficiency on expression of PKC-ϵ and this revealed strong upregulation of this negative regulator (Fig. 5). Further support for an interacting network of PKC signalling is provided by the finding that, reflecting the profound suppression of antigen-stimulated cytokine production in these cells, PKC-α expression is strongly downregulated in PKC-θ-deficient cells perhaps suggesting there is coordinate regulation of these cytokine-promoting PKC isoforms in BMMCs (Fig. 5). Collectively, these data may suggest that by downregulating PKC-α, ES-62 both disrupts cytokine-promoting pathways and induces PKC-ϵ-mediated cytokine-suppressing pathways (Fig. 6).

## 4 Discussion

Exposure to antigen results in rapid FcϵRI-dependent mast cell degranulation of pre-formed mediators such as cytokines, histamine, prostaglandins and leukotrienes and the de novo synthesis of cytokines [22], [23], responses that contribute to the symptoms and clinical manifestations of allergic disorders such as asthma [24]. The parasitic worm-derived immunomodulator, ES-62 induces mast cell hyporesponsisveness and can directly interfere with mast cell-dependent hypersensitivity in the skin and lungs by blocking key FcϵRI- and TLR4-coupled signals, such as calcium mobilization and PKC-α activation [10], [13]. Our previous work on human BMMCs showed that ES-62 interacted with TLR4 at the cell surface and this lead to internalization of the complex followed by sequestration (by an unknown mechanism) and subsequent degradation of PKC-α via a caveolae-lipid raft, non-proteosomal mechanism [13]. However, ES-62 also causes degradation of PKC-ζ in human mast cells but this is via a ubiquitin-proteosome-dependent mechanism. Nevertheless, our studies on different mouse-derived mast cell subsets indicate that both mechanisms of degradation can operate with respect to PKC-α [10] indicating the complexity of the process. Further studies on different mast cell types with respect to PKC isoform expression and function may therefore be warranted. Additional support that down-regulation of PKC-α plays a significant role in the ES-62-mediated hyporesponsiveness of mast cells, is now provided by our studies using BMMCs from PKC-α-deficient mice, that demonstrate important but counter-regulatory roles for this PKC isoform in FcϵRI- and LPS/TLR-4 mediated IL-6 production. Whilst our findings that PKC-α promotes FcϵRI-responses are consistent with those from other groups (e.g. Ref 14), the differential effects on FcϵRI/TLR4 signalling were unexpected, as a previous study in BMMCs deficient in PKC-α, -β or indeed both isoforms, suggested that such conventional isoforms did not modulate LPS signalling in mast cells [25]. Interestingly, this study did not find PKC-α to be important for FcϵRI-mediated IL-6 production either but as the authors measured cytokine release after 3 h this may reflect that this PKC isoform exerts its effects on the de novo synthesis of cytokines, rather than on exocytosis of preformed mediators. Likewise, PKC-β isoforms have been reported to promote FcϵRI-mediated IL-6 release [14], [16], [25] whereas our data suggest PKC-β acts to suppress both FcϵRI and LPS-stimulated production, discrepancies that may again be reconciled by the potential differential regulation of degranulation of preformed cytokines and de novo cytokine synthesis and secretion. Moreover, the lack of IL-6 production in the PKC-β^−/−^ cells witnessed by Nechushtan was overcome when the sensitisation step with IgE was increased from 2 h to 4 days, suggesting that FcϵRI ligation by IgE alone modulates mast cell signals and in our experiments we sensitise with IgE overnight. Indeed, we have previously shown such IgE sensitisation to amplify LPS/TLR4 responses in mast cells [10]. Additionally, the consequence of IgE-mediated mast cell activation is not only dependent upon the amount of specific IgE present, but it can also be modulated by the dose of the cross-linking antigen. Despite WT and KO BMMCs showing similar behaviour, Lessmann et al., witnessed differential IL-6 release from both cell types when these cells were stimulated with two different concentrations of antigen. Interestingly, the lower concentration of DNP-HSA (20 ng/mL) employed in the study provoked larger amounts of cytokine release in comparison to the higher concentration (200 ng/mL) [26]. Thus, it seems likely that the role of a PKC isoform is determined by the response provoked by an allergen/stimulus and that this role may alter depending on which signalling pathway i.e. whether it is the immediate inflammatory response (exocytosis) or the more sustained response (de novo synthesis of cytokines) is activated. It would appear that the role of a PKC isoform is therefore context dependent; similar to the functional responses of mast cells depending upon the stimuli they receive [10].

Although, and consistent with the idea that ES-62 mediates at least some of its desensitising effects via downregulation of PKC-α, FcϵRI-mediated IL-6 and TNF-α production was substantially reduced in PKCα-deficient BMMCs and ES-62 could further suppress the residual activity. This possibly reflected that ES-62 can additionally downregulate PKC-δ in BMMCs [10] and this isoform has also been shown to promote antigen-stimulated mast cell responses [27], [28]. However, PKC-ϵ and θ are also expressed by BMMCs [14] and so we investigated their potential role in ES-62-mediated BMMC hyporesponsiveness. Like PKC-α, and as reported previously for RBL cells [29], we found PKC-θ to play an important role in FcϵRI-mediated cytokine production in BMMCs, as indicated by PKC-θ^−/−^ cells releasing substantially reduced levels of both IL-6 and TNF-α. However, in contrast to what was observed in PKC-α-deficient cells, although ES-62 could still slightly suppress the residual cytokine production by PKC-θ^−/−^ BMMCs, essentially all of the inhibition observed in WT cells could be accounted for by that resulting from PKC-θ deficiency suggesting that this isoform may be an important target, either directly or indirectly in BMMCs. Of note, it has previously been reported that PKC-θ^−/−^ T cells show a defective response to activation, which can lead to increased apoptosis [30] but we found no evidence of increased apoptosis of PKC-θ^−/−^ BMMCs relative to WT control cells following FcϵRI-mediated cross-linking or LPS exposure as measured by 7-AAD staining (results not shown). Thus, the deceased cytokine responses observed with PKC-θ^−/−^ BMMCs is not due to cell death.

To date, the role of PKC-ϵ in mast cell biology has been rather controversial with one study implicating a redundant role for this isoform, as the loss of PKC-ϵ had no impact on IL-6 or TNF-α production [26]. However, our data are suggestive of this novel isoform being a negative regulator of mast cell effector function following antigen stimulation as we have shown that IL-6 production was significantly up-regulated in the KO cells stimulated via FcϵRI. Interestingly therefore, two independent studies in RBL cells have previously supported a role for PKC-ϵ as a negative regulator of signalling events such as calcium mobilization and/or MAP kinase activation [15], [31], and we have shown ES-62 to suppress FcϵRI-mediated calcium mobilisation in both human and mouse mast cells [10], [13]. Of note, despite the increased IL-6 production in the PKC-ϵ^−/−^ cells, ES-62 was still able to decrease the levels of the cytokine.

Finally, as our studies suggest that PKC-α and θ can play redundant or similar roles in promoting FcϵRI-mediated responses that can potentially be counter-regulated by PKC-β and/or -ϵ, we investigated whether ES-62 may exert its desensitising effects, either directly or indirectly, by targeting this PKC network, by determining the effect of PKC-α-deficiency on PKC-ϵ expression. Western blot studies showed that PKC-α^−/−^ BMMCs have substantially elevated expression of PKC-ϵ in comparison to their wild-type counterparts raising the possibility that by directly acting to downregulate the cytokine promoting effects of PKC-α, ES-62 may in a compensatory manner additionally upregulate a negative regulator, PKC-ϵ, to more efficiently inhibit mast cell IL-6 cytokine responses. We also tried to extend this type of analysis to the other PKC isoforms but unfortunately could not detect them by blotting in BMMC samples in spite of using several different antisera for each isoform and successfully detecting them in appropriate positive control cells in each case. We can only conclude that these isoforms are expressed in our BMMCs from different genetic backgrounds at levels that are below the levels of detection of the indicator system employed in the blotting.

## Figures and Tables

**Table 1:** Development of BMMCs in vitro from PKC isoform^−/−^ and matched WT mice: c-kit and FcϵRI expression levels analysed by flow cytometry for PKC– α, -β, -ϵ and –θ.

PKC isoform	% c-Kit+ FcϵRI+ cell WT	% c-Kit+ FcϵRI+ cell KO
PKC-α	86.0	92.0
PKC-β	87.5	85.0
PKC-ϵ	87.0	95.5
PKC-θ	94.8	96.8

**Table 2:** Cytokine responses of BMMCs in response to antigen mediated cross-linking of FcϵRI or stimulation with LPS as summarised by the percentage (%) of the PKC isoform deficient BMMCs in comparison to their WT counterpart. Data are presented as the mean value ± SEM from at least 3 independent experiments, except TNF-α release by matched WT and PKC-θ^−/−^ BMMCs following LPS stimulation where the data presented are representative of two experiments and thus the mean is displayed only. ND = not detected and – represents no change.

	PKC-α	PKC-θ	PKC-β	PKC-ϵ
IL-6: FcϵRI	71 ± 14%	20 ± 10%	152 ± 21%	158 ± 7%
IL-6: LPS	159 ± 8%	30 ± 9%	158 ± 4%	–
TNF-α: FcϵRI	55 ± 10%	11 ± 7%	ND	ND
TNF-α: LPS	–	30%	162 ± 5%	–
